# The low density receptor-related protein 1 plays a significant role in ricin-mediated intoxication of lung cells

**DOI:** 10.1038/s41598-020-65982-2

**Published:** 2020-06-02

**Authors:** Reut Falach, Anita Sapoznikov, Yoav Gal, Eytan Elhanany, Yentl Evgy, Ohad Shifman, Moshe Aftalion, Sharon Ehrlich, Shlomi Lazar, Tamar Sabo, Chanoch Kronman, Ohad Mazor

**Affiliations:** 1 Department of Biochemistry and Molecular Genetics, Israel Institute for Biological Research, 19 Reuven Lerer St., Ness-Ziona, 76100 Israel; 2 Department of Pharmacology, Israel Institute for Biological Research, 19 Reuven Lerer St., Ness-Ziona, 76100 Israel; 3 Department of Infectious Diseases, Israel Institute for Biological Research, 19 Reuven Lerer St., Ness-Ziona, 76100 Israel

**Keywords:** Cell biology, Biochemistry

## Abstract

Ricin, a highly lethal plant-derived toxin, is a potential biological threat agent due to its high availability, ease of production and the lack of approved medical countermeasures for post-exposure treatment. To date, no specific ricin receptors were identified. Here we show for the first time, that the low density lipoprotein receptor-related protein-1 (LRP1) is a major target molecule for binding of ricin. Pretreating HEK293 acetylcholinesterase-producer cells with either anti-LRP1 antibodies or with Receptor-Associated Protein (a natural LRP1 antagonist), or using siRNA to knock-down LRP1 expression resulted in a marked reduction in their sensitivity towards ricin. Binding assays further demonstrated that ricin bound exclusively to the cluster II binding domain of LRP1, *via* the ricin B subunit. Ricin binding to the cluster II binding domain of LRP1 was significantly reduced by an anti-ricin monoclonal antibody, which confers high-level protection to ricin pulmonary-exposed mice. Finally, we tested the contribution of LRP1 receptor to ricin intoxication of lung cells derived from mice. Treating these cells with anti-LRP1 antibody prior to ricin exposure, prevented their intoxication. Taken together, our findings clearly demonstrate that the LRP1 receptor plays an important role in ricin-induced pulmonary intoxications.

## Introduction

Ricin toxin is a heterodimeric molecule comprising two polypeptide chains, A (RTA) and B (RTB). RTA, the catalytically active subunit, depurinates a single adenosine within the GAGA sequence in the sarcin-ricin loop of the 28 S rRNA molecule^1,2^, thereby inhibiting protein synthesis, an event leading to cell death^3^. Toxin cell entry is effectuated by the RTB lectin chain, a member of a carbohydrate-binding protein family that exhibits highly specific binding to sugar moieties within a larger carbohydrate or as a part of glycoprotein/glycolipid molecules. In the case of ricin, the lectin-domains of RTB exhibit binding specificity for terminal galactose and N-acetyl-galactosamine residues^4–6^. Common knowledge states that ricin can bind effectively to any glycol-conjugate that contains galactose residues. Hence, each of the numerous cell surface glycoproteins or glycolipids that contain galactose can serve as a receptor for ricin binding and intracellular uptake. However, previous studies at our laboratory showed that following pulmonary exposure of mice to ricin, the toxin displays differential patterns of binding with various lung-cell populations and that ricin-induced depurination levels in these cells differed markedly^7,8^. These findings, which suggest that ricin enters different cells in a selective manner led us to investigate whether the ricin molecule binds to specific cell-surface receptors.

In the present study, mass spectrometry analysis identified the transmembrane receptor low-density lipoprotein receptor related-protein 1 (LRP1) as a component of complexes formed between ricin and mice lung membrane proteins. The role of ricin-LRP1 interactions in ricin-mediated cytotoxicity was probed by determining the effect of functional blocking of LRP1 on ricin-induced cytotoxicity. We show that prevention of ricin binding to this receptor is sufficient to protect cultured cells as well as primary lung cells from intoxication.

These findings demonstrate for the first time that the transmembrane receptor LRP1 plays a central role in ricin poisoning and open new vistas for the development of novel therapeutic agents for dealing with ricin-induced intoxications.

## Results

### Interactions of ricin with membrane-bound proteins from mice lungs

**Figure 1:**
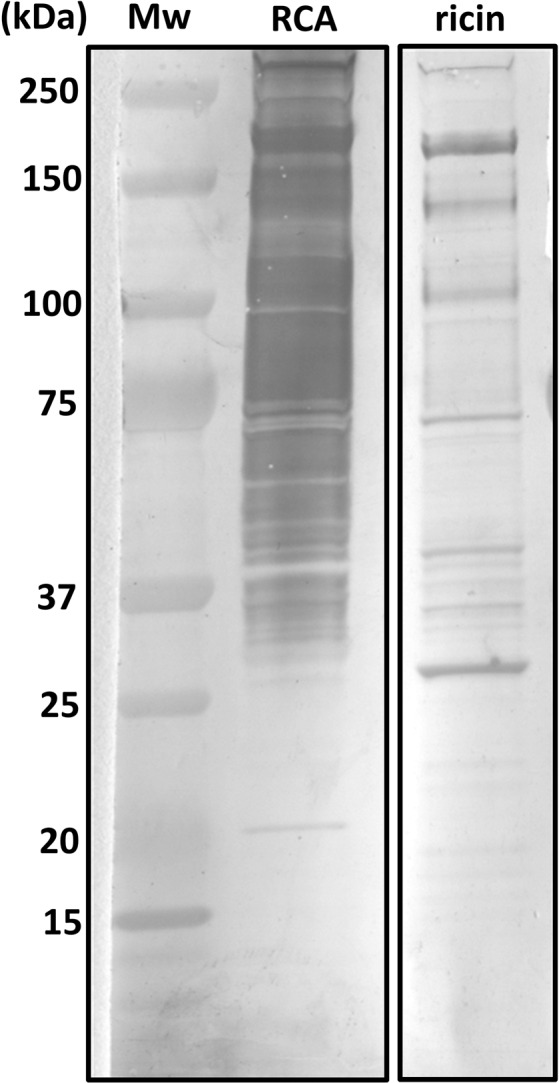
Lectin blot of membrane-bound proteins from mice lungs: Lung cell membrane proteins were resolved by SDS-PAGE, transferred to absorbent membranes, and incubated with purified preparations of RCA or ricin. Black frames indicate that these are non-consecutive lanes taken from two blots.

To examine whether ricin binds in a differential manner to cell-surface proteins, murine lung cell membrane proteins were resolved by SDS-PAGE and transferred to absorbent membranes which were then incubated with purified preparations of either ricin or ricin-related *Ricinus communis* agglutinin (RCA). Labeling with polyclonal anti-ricin antibody, which interacts with both ricin and RCA, revealed that while RCA seems to bind in an indiscriminate manner to a wide range of lung cell membrane proteins, purified ricin was found to bind to a limited number of discrete protein bands (Fig. 1).

### Identification of ricin-bound lung cell membrane proteins

The labeled bands detected above, consist of proteins which were extracted from lung cell outer membranes and then resolved by SDS-PAGE and electro-transferred to a PVDF membrane. These processes are expected to alter the conformational structures of respective proteins in a radical manner and therefore their apparent *in-vitro* interaction with ricin may not reflect faithfully the binding that occurs between ricin and cell-membrane bound proteins in intact cells. To redress this issue, ricin was allowed to interact with lung cell membranes and proteins were then resolved on native gels under conditions which are expected to preserve protein/ricin complexes intact. Protein transfer was also performed under unique conditions to avoid protein complex disruption, utilizing the Blue-native polyacrylamide gel electrophoresis (BN-PAGE) methodology^9^. Following labeling with polyclonal anti-ricin antibodies, 3 faint high molecular weight bands (~480–720 kDa) were discerned. These were excised, destained and processed by In-gel digestion (reduction, alkylation and digestion) and then subjected to mass spectrometric analysis.

Sequence analysis of the 3 bands led to identification of ricin in conjunction with either mannose receptor (band #1) or low-density lipoprotein receptor-related protein 1 (LRP1, band #2 and #3) (Table 1). Binding of ricin to the mannose receptor, has been reported in the past^10,11^, however, expression of this receptor is confined to a relatively small number of cell. Unlike the mannose receptor, LRP1 is highly distributed in cells and tissues yet its interaction with ricin and thereby its possible role in toxin uptake has not, to the best of our knowledge, been documented.

**Table 1:** Mass spectrometry identification of ricin-associated proteins.

Band #	Description	Coverage	Unique peptides	PSMs
**1**	Mannose-receptor	21.9	32	253
**2**	Prolow-density lipoprotein receptor-related protein 1	17.8	63	264
**3**	Prolow-density lipoprotein receptor-related protein 1	25.9	88	331

Data is an average of two analysis assays; Masscot analysis and Sequest analysis. **Coverage**- the percent of protein sequence that is covered by the identified peptides. **Unique peptides-** number of identified peptides that are specific to this protein. **PSMs -** equilibrium value of the number of identified peptides with the number of their repeats in the sample.

### Confocal microscopy analysis of ricin binding to LRP1 in cultured HEK293 cells

**Figure 2:**
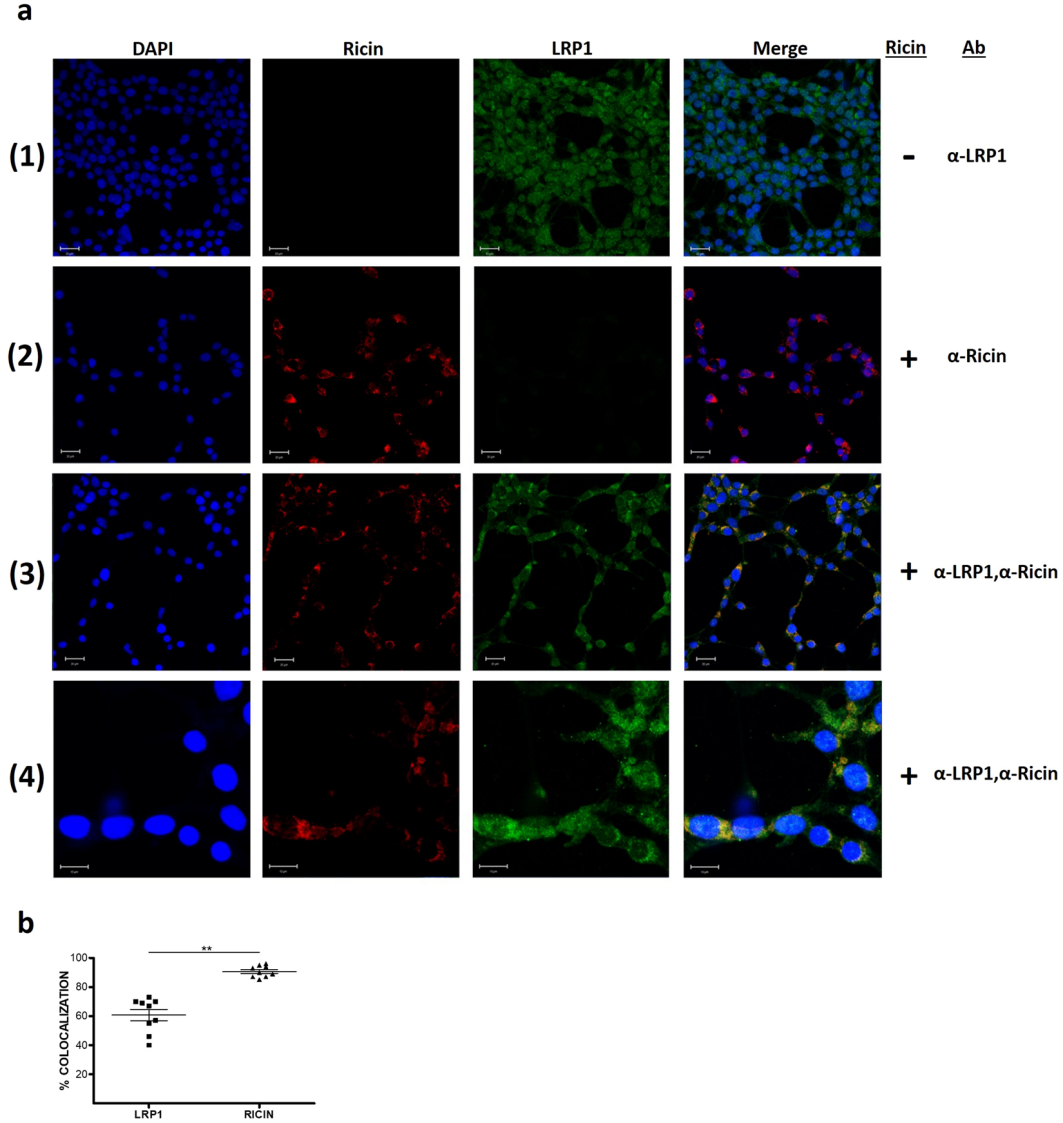
Immunostaining and confocal fluorescence microscopy of HEK293 cells. (**a**) HEK293 cells were incubated with ricin, fixed and then immunostained with anti-ricin (Alexa Fluor 594, red) and anti-LRP1 (Alexa Fluor 488, green) antibodies. Nuclei were stained with DAPI (blue). Rows 1 and 2 display single-fluorophore controls for each antibody. Insets in the right-hand column of rows 3 and 4, highlight the cellular localization of ricin and LRP1 complexes (yellow staining). Data are representative of images obtained from triplicate independent experiments with similar results. (Scale bars: lines 1–3, 20 µm; line 4, 20 µm. Magnifications: lines 1–3, ×400; line 4, ×1600). (**b)** Quantification of the percent of the co-localization for each protein (LRP1 or ricin) from the total immunostained protein by the Zen software (version 2.1, 2008; Zeiss). Results of 9 measurements with mean ± SE.

To characterize the binding of ricin to LRP1, the localization of these two proteins in ricin-intoxicated HEK293 cells was determined. To this end, HEK293 cells were grown to confluence and then incubated with ricin. Cells were then fixed, stained simultaneously with fluorescently-labeled anti-ricin and anti-LRP1 antibodies and visualized by immunofluorescence confocal microscopy. As shown in Fig. 2a, merging of the anti-ricin and anti-LRP1 stained cells resulted in highly co-localized staining (yellow staining, rows 3–4, right panel). Rows 1 and 2 in Fig. 2a display single-fluorophore controls for each antibody. Quantification of % co-localization showed that 90.6% of the ricin is linked to LRP1 and that 60.7% of the LRP1 is occupied by ricin (Fig. 2b), suggesting that LRP1 has a major and nearly-exclusive contribution to the binding of ricin to the outer-surface of these cells.

### Blocking LRP1 abrogates ricin toxicity in HEK293 cells

**Figure 3:**
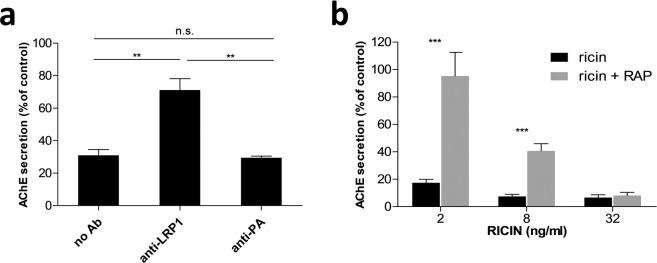
Anti-LRP1 antibody and RAP reduce ricin-induced cytotoxicity of HEK293 cells. (**a**) Cells were treated with rabbit-anti-LRP1 antibody 1 hour prior to ricin (10 ng/ml) intoxication. 18 hours after intoxication the secretion of AChE was quantified. Results summarize 3 measurements with mean ± SE. (**b)** Cells were pretreated with RAP (10 nM) and then exposed to increasing doses of ricin (2–32 ng/ml) at 4 °C. Cells were then washed and incubated at 37 °C for 24 h after which secreted AChE levels were quantified. Values represent mean ± SE of 3 independent measurements.

To assess the contribution of LRP1 to ricin-mediated cytotoxicity, we examined whether preclusion of ricin binding to LRP1 would protect cells from ricin intoxication. To this end, we utilized the genetically engineered HEK293-AChE cell line, which constitutively synthesizes and secretes large amounts of acetylcholinesterase (AChE) to the culture medium^12^. Ricin mediated protein synthesis arrest, an early event in ricin intoxication, results in diminished production and secretion of AChE with a half maximal inhibitory concentration (IC_50_) of 0.1 ng/ml. We first examined the effect of anti-LRP1 antibodies on HEK293 ricin-induced intoxication. To this end, cells were pre-incubated with rabbit-anti-LRP1 antibody, exposed to ricin (10 ng/ml, 100 IC_50_) for 1 hour, rinsed and incubated at 37 °C for 18 hours, after which secreted AChE levels were quantified. Cells which were not pre-incubated with anti-LRP1 antibody, served as a positive control for ricin intoxication. While exposure of the control cells to ricin led to a 70% reduction in secreted AChE levels, pretreatment with anti-LRP1 antibodies led to significantly higher levels of secreted AChE, which were only 30% lower than the levels exhibited by non-intoxicated cells. Pretreatment of the HEK293-AChE cells with a non-related antibody (anti-*B. anthracis*-PA antibody) did not protect cells from intoxication; the extracellular levels of AChE measured in these cells were as low as those measured in ricin-intoxicated cells that were not treated with antibodies (Fig. 3a).

For specific binding-site blockage of the LRP1 receptor, we utilized Receptor-Associated Protein (RAP), a natural antagonist of LRP1^13^. We tested RAP’s ability to competitively inhibit binding and entry of ricin into HEK293-AChE cells. To this end, cells were chilled to 4 °C and incubated with RAP. These conditions are compatible with ligand-receptor binding yet do not allow internalization of the newly formed ligand/receptor complexes. After 1 hour ricin was added at different concentrations for 1 hour, then cells were washed and incubated at 37 °C for 24 hours to allow internalization of cell-linked ricin. Cells exposed to ricin without preincubation with RAP served as a positive control for ricin intoxication. As shown in Fig. 3b, cells exposed to ricin at a concentration of 2 ng/ml (20 IC_50_) displayed ~80% decrease in secreted AChE activity, while cells which were treated with RAP prior to the addition of the same concentration of toxin expressed nearly normal levels of AChE (95% compared to non-intoxicated cells). Only when the cells were incubated with higher ricin concentrations was RAP-related protection compromised in a dose dependent manner. Thus, when the cells were exposed to a 4-fold higher dose of ricin (8 ng/ml, 80 IC_50_), pre-incubation with RAP resulted in 40% AChE activity compared to 7.5% activity without RAP pretreatment, while exposure to a 16-fold higher dose of ricin (32 ng/ml, 320 IC_50_), abolished the beneficial effect of RAP. In this latter instance, both RAP-pretreated and non-treated cells displayed no more than residual levels of secreted AChE levels (8.7 and 7.1% respectively). Taken together, this set of experiments confirms that the LRP1 receptor plays an important role in ricin-induced intoxication and that functional antagonism of the LRP1 receptor leads to substantially reduced sensitivity of the cells to ricin.

### Reduced toxicity of ricin in HEK293 cells following transient knockdown of LRP1

**Figure 4:**
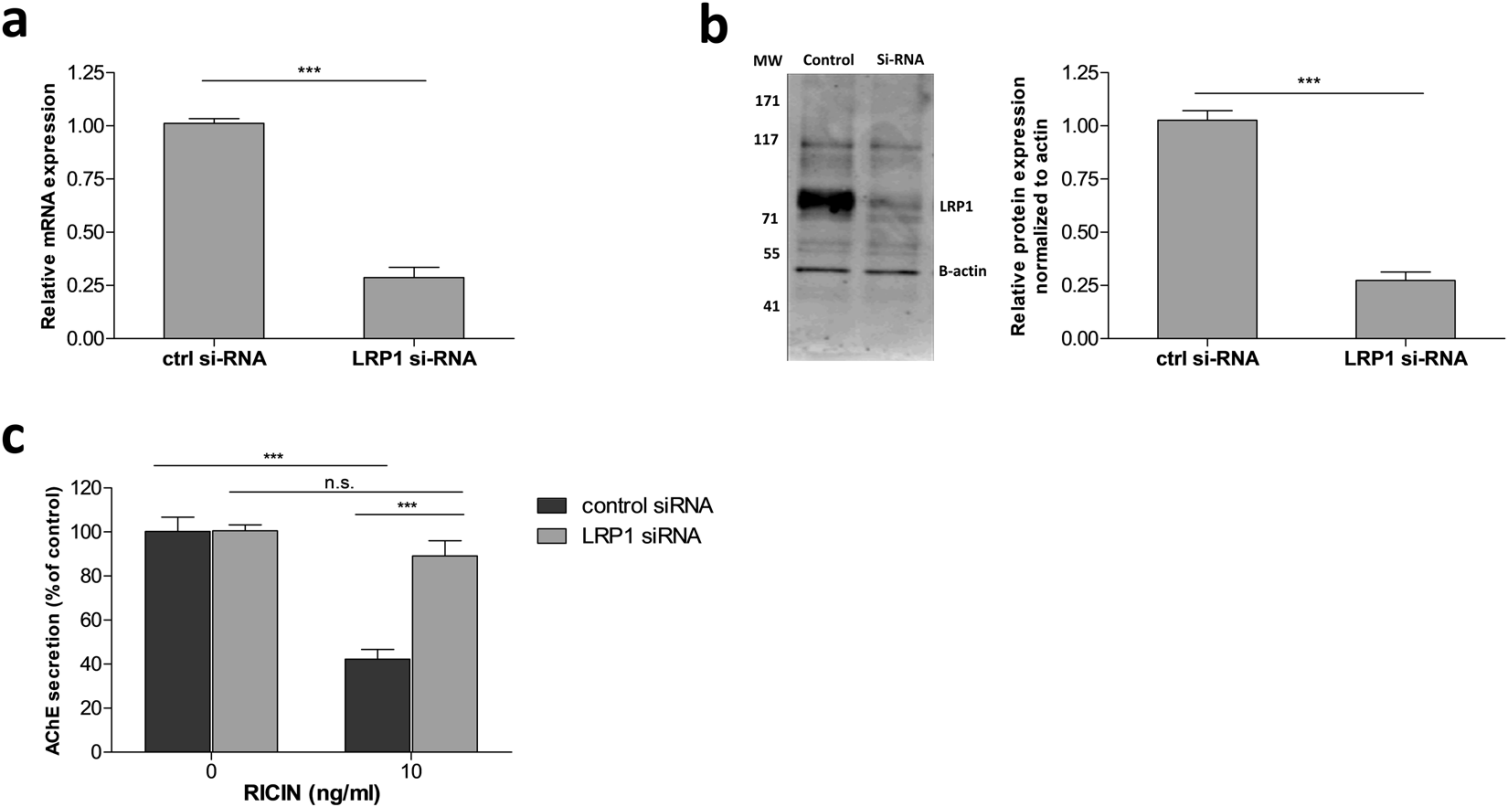
LRP1 silencing in HEK293-AChE cells. Cells were transfected with LRP1 siRNA or control siRNA using Lipofectamine. After 48 hour of transfection, cells were harvested for western blotting/RNA extraction, or intoxicated with ricin. (**a**) mRNA levels of LRP1 were quantified by real-time PCR after treatment of the cells with control or LRP1 siRNAs. Results are presented as mean ± SEM of 3 measurements. (**b**) Western blotting analysis for the control or LRP1 siRNAs treatments using rabbit derived anti-LRP1 antibodies. Anti-actin blot was used as a loading control. Densitometric analysis of Western blots from triplicate samples was performed. (**c**) Cells treated with control or LRP1 siRNAs were exposed to ricin (10 ng/ml) and 18 hours later secretion of AChE was quantified. Results are presented as mean ± SE of 3 measurements.

To confirm the role of LRP1/ricin interactions in ricin intoxication, we examined whether reduction of LRP1 expression affects cell sensitivity towards the toxin. To this end, LRP1 expression was knocked-down by LRP1 siRNA. Under the conditions at which these experiments were held, 48 hours after transfection of HEK293-AChE cells with LRP1 siRNA, gene knockdown efficiency was ~70% as determined by RT-PCR (Fig. 4a), and protein knockdown efficiency was ~75% as determined by Western blot analysis (Fig. 4b). The LRP1 knocked-down cells were then exposed to ricin (10 ng/ml) for 1 hour, rinsed and then incubated at 37 °C for 18 hours after which AChE secretion was quantified. Reduction of LRP1 expression led to decreased cytotoxicity following ricin intoxication. Thus, while ricin exposure of cells with normal levels of LRP1 led to markedly reduced secreted AChE levels, equivalent to ~40% of that displayed by the non-intoxicated control cells, partially LRP1 knock-down cells that were exposed to the toxin at the same concentration, displayed considerably higher levels of secreted AChE, equivalent to 89% of AChE secretion exhibited by the non-intoxicated LRP1 knocked-down cells (Fig. 4c). Taken together, three different experimental approaches clearly indicate that the LRP1 receptor plays a dominant role in determining ricin cytotoxicity.

### Ricin binds to LRP1 binding-cluster II

**Figure 5:**
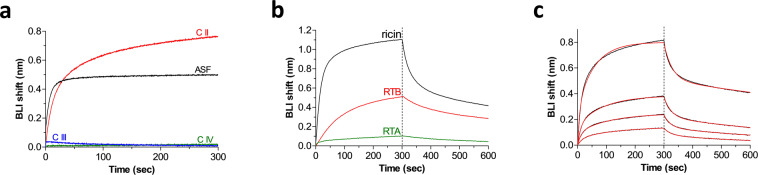
Binding of ricin to LRP1 clusters. (**a**) Streptavidin-biosensors coated with biotinylated LRP1 cluster II (C II, red), LRP1 cluster III (CIII, blue), LRP1 cluster IV (C IV, green) or asialofetuin (ASF, black) were submerged in wells containing ricin (10 µg/ml) and the wavelength interference was recorded. (**b)** Streptavidin-biosensors coated with biotinylated C II were submerged for 300 seconds in wells containing: ricin (black), RTB (red) or RTA (green). (**c**) Increasing concentrations of ricin (black lines; from bottom up: 42 nM, 83 nM, 167 nM and 330 nM). Red lines: curve fitting of the 2:1 binding model. The sensors were then immersed in buffer for another 300 seconds (dissociation phase).

The extra-cellular segment of LRP1 comprises four complement-type repeats (CRs) that are further divided to four distinct clusters (I-IV) to which most of the known LRP1 ligands bind^14^. It was therefore of interest to determine which cluster of LRP1 serves as the binding site of ricin. To this end, biotinylated-LRP1 clusters II-IV, known to bind most of the identified LRP1 ligands, were separately immobilized on Octet streptavidin-biosensors and interacted with ricin toxin. Biotinylated asialofetuin (ASF), a well-known ligand of ricin, was also immobilized on Octet streptavidin-biosensors and served as a positive control for ricin binding. Only LRP1 cluster II was found to bind ricin, whereas no measurable interactions between ricin molecules and LRP1 cluster III or IV could be discerned (Fig. 5a).

To examine whether the binding of ricin to LRP1 cluster II indeed represents a *bona fide* RTB-driven interaction, we measured binding rates of ricin and its isolated subunits to biotinylated soluble cluster II on an Octet sensor. When ricin holotoxin (10 μg/ml) was added, it quickly bound to cluster II, reaching near saturation at about 1 nm shift and dissociated in a bi-phasic manner (Fig. 5b). Next, the cluster II-biosensor was interacted with purified RTB (10 μg/ml) inducing a marked wavelength interference reaching about 0.5 nm after 300 seconds. As the wavelength shift is proportional to the protein mass, these results fit well with the fact that the molecular weight of RTB is approximately half of the holotoxin (33 kDa and 67 kDa, respectively). In contrast, when cluster II interacted with a purified preparation of the catalytic A subunit of ricin (RTA, 10 μg/ml), low-to-insignificant binding was observed (the residual binding probably reflects impurities of holotoxin in the RTA preparation, which are estimated to be less than 5%).

The binding kinetics of ricin to cluster II were characterized using the same platform with increasing concentrations of ricin. As ricin has two nearly identical lectin-binding site located within its B-subunit, it was assumed that each binding site will bind the receptor independently. Accordingly, the binding sensograms were fitted using the 2:1 heterogeneous ligand model which is a combination of two 1:1 curve fits. Indeed, this model resulted in an excellent fit to the binding sensograms for the tested ricin concentrations (r = 0.99, Fig. 5c). Conversely, when the binding data was fitted using a model in which ricin binds LRP1 at only one site (1:1), a poor fit was generated. Using the 2:1 model, the overall affinities values (K_D_) of the two binding sites of ricin toward LRP1 were calculated to be 81 and 47 nM (K_D_1 and K_D_2, respectively). These results support the assumptions that the two ricin-lectin binding sites interact with LRP1 in an independent manner, albeit, at similar affinities.

### The effect of ricin-neutralizing antibodies on the interaction with LRP1

**Figure 6:**
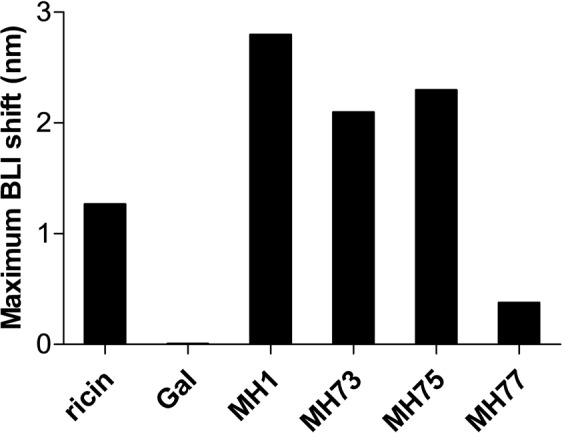
Binding of ricin to cluster II in the presence of neutralizing antibodies. Streptavidin-biosensors coated with biotinylated C II were submerged for 300 seconds in wells containing ricin and either galactose or the indicated antibodies and the maximal wavelength interference was determined.

Previously, phage-display libraries based on antibody-encoding genes originated from ricin-immunized non-human primates, were utilized to isolate a set of anti-ricin monoclonal antibodies which bind to either RTA or RTB^15^ and their ability to neutralize ricin was demonstrated both *in vitro* and *in vivo*^16^. In view of our findings regarding the role of LRP1 in the intoxication process of ricin, it was of interest to determine whether one or more of these monoclonal neutralizing antibodies impairs the binding of ricin to LRP1. To address this issue, biotinylated cluster II was immobilized on an Octet biosensor and the maximal wavelength interference induced by ricin was evaluated in the absence or the presence of each antibody. To set up the experimental system, the binding of ricin was first tested in the presence of excess of galactose which was shown before to bind the lectin-binding moieties of the toxin. As expected, while ricin induced a wavelength shift of about 1.3 nm, galactose completely abolished the binding of the toxin to cluster II (Fig. 6).

Next, ricin was pre-incubated with each antibody and each of the formed toxin/antibody complexes was interacted with the immobilized cluster II. We first tested the MH1 mAb which targets RTA and found that the ricin-MH1 complex induced a significant increase in the wavelength shift compared to that of ricin alone (Fig. 6). As the extent of the wavelength interference is dependent in part upon the antigen mass, these results fit well with the assumption that the binding of MH1 to ricin does not hamper RTB-mediated binding to the receptor and since the mass of the MH1-ricin complex is larger than that of the toxin alone, the net result is an increase in the apparent signal.

The monoclonal antibodies MH73, MH75 and MH77 bind with similar affinities to non-overlapping epitopes located on the surface of RTB^15^. Formation of complexes between ricin and the MH73 or MH75 mAbs did not prevent the toxin binding to cluster II and actually, once again, increased the measured wavelength shift (Fig. 6). In contrast, antibody MH77 reduced the binding of ricin to cluster II by more than 70%.

These results suggests that the MH77 anti-ricin monoclonal antibody and cluster II of LRP1 interact with the same region/epitope of ricin. It may well follow that the neutralizing effect of MH77 is due to its ability to prevent ricin binding to the LRP1 receptor.

### Role of the LRP1 in ricin induced cytotoxicity of lung cells

**Figure 7:**
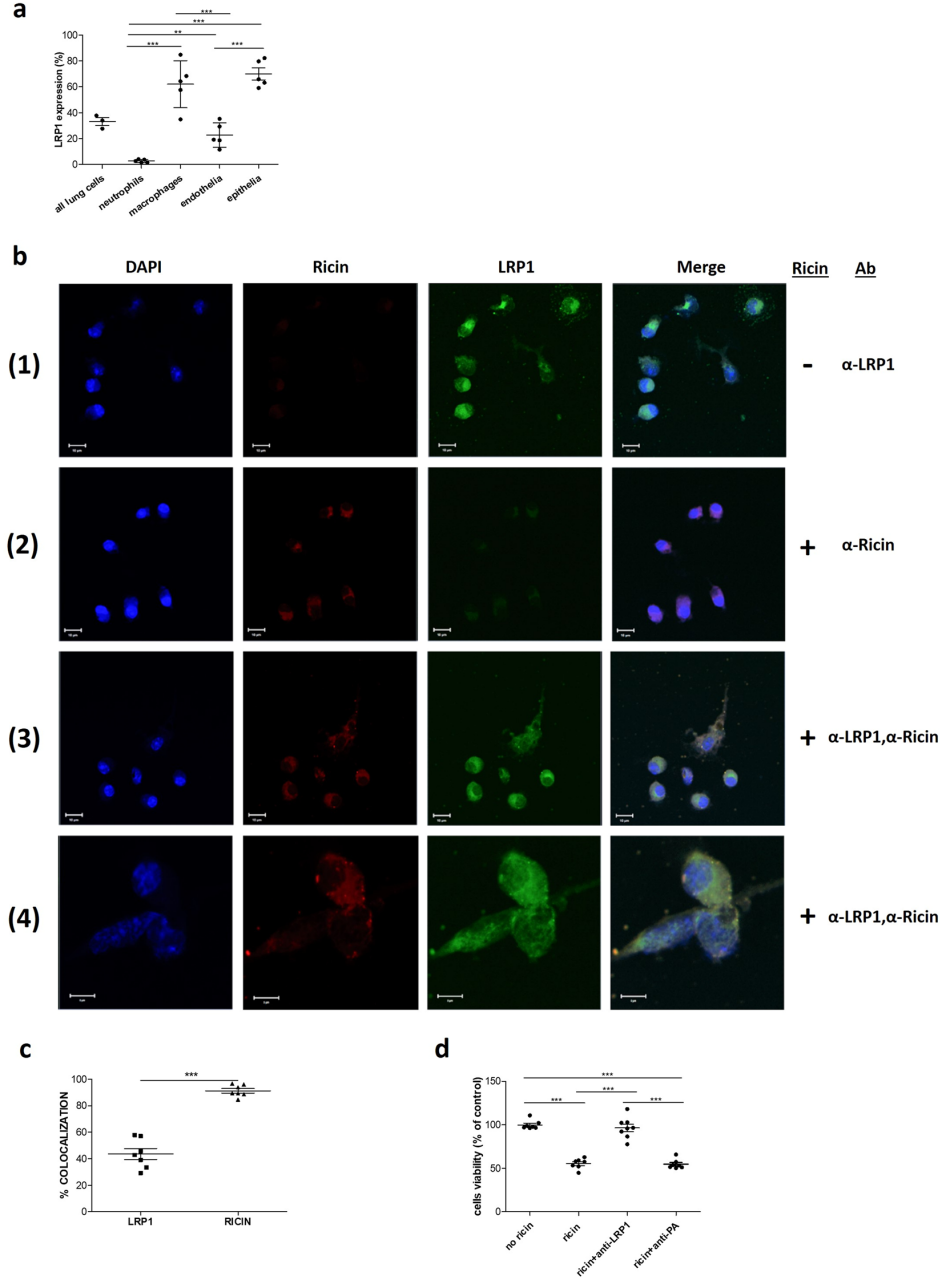
LRP1 role in ricin induced cytotoxicity of lung cells. (**a**) Flow cytometry analysis of LRP1 expression in different lungs cell populations. SCSs were produced from lungs of naïve mice (n = 3–5), separated, stained for neutrophils, macrophages, endothelial and epithelial cells and anti-LRP1 antibody for measurement LRP1 expression in differential cell types. (**b**) Immunostaining and confocal fluorescence microscopy of lung SCSs. SCSs were incubated with ricin, fixed and then immunostained with anti-ricin (Alexa Fluor 594, red) and anti-LRP1 (Alexa Fluor 488, green) antibodies. Nuclei were stained with DAPI (blue). Rows 1 and 2 display single-fluorophore controls for each antibody. Insets in the right -hand columns of line 3 and 4, highlight the cellular localization of ricin and LRP1 complexes (yellow staining). Data are representative of images obtained from triplicate independent experiments with similar results. (Scale bars: lines 1–3, 10 µm; line 4, 5 µm. Magnifications: lines 1–3, ×800; line 4, ×2400). (**c**) Quantification of the percent of the co-localization for each protein (LRP1 or ricin) from the total immunostained protein by the Zen software (version 2.1, 2008; Zeiss). Results of 7 measurements with mean ± SE. (**d**) Lung SCSs were pretreated with anti-LRP1 or anti-PA antibody 1 hour prior to ricin exposure (10 ng/ml) and cells viability was assessed by the XTT assay at 48 hours post exposure (n = 7).

In the series of experiments described above, the contribution of LRP1 receptor to ricin intoxication was examined in cultured cells (HEK293-AChE). It was therefore of interest to examine whether LRP1 plays a similarly significant role in ricin-mediated cytotoxicity in primary cells. Ricin intoxication is considered most toxic *via* the pulmonary route of exposure. We have previously reported that following pulmonary exposure of mice to ricin, different lung cell types bind the toxin at different rates and levels^7^. Moreover, we found that the ricin-mediated rRNA depurination process occurs in different cell populations (24 hours post exposure) at distinctly different levels^8^. Thus, depurination in neutrophils was negligible, while macrophages and endothelial cells displayed 10.8% and 22% depurination values, respectively. The most pronounced depurination activity was measured in epithelial cells, where depurination was found in more than 80% of these cells 24 hours post-exposure to ricin. In view of the role of LRP1 in ricin intoxications described above, we examined whether a correlation exists between the levels of depurination and LRP1 expression in these different lung cell types. To this end, single cell suspensions (SCSs) of mice lungs were subjected to flow cytometric analysis with both anti-cell-type specific and anti-LRP1 antibodies. While the overall expression of LRP1 on lung cells was 33% (33 ± 2.9% of the cells were LRP1 positive), neutrophils displayed no more than near-to background levels of LRP1 (2.7 ± 0.53), while 22.8 ± 4.2 and 70 ± 4.6 percent of the lung endothelial and epithelial cells respectively, expressed this membrane-bound receptor, in excellent correlation to their measured depurination levels following ricin intoxication (Fig. 7a). In contrast, the high level of LRP1 expression in lung macrophage cells, 62 ± 8.1%, did not correlate with their measured depurination levels (~11%). We note however, that unlike the other lung cell types examined, macrophages are eliminated very rapidly from the lungs following ricin exposure^7^ so that their depurination levels, determined at 24 hours post exposure long after removal of most of the macrophages, is a gross underestimation of the actual depurination process in this cell type.

To test whether ricin toxin binds the lungs cells *via* the LRP1 receptor, lung SCS was exposed to ricin (10 ng/ml) for 24 hours at 37 °C and then fixed and stained simultaneously with specific antibodies directed against ricin and LRP1 and visualized by immunofluorescence confocal microscopy. As shown in Fig. 7b, merging of the anti-ricin and anti-LRP1 stained cells resulted in yellow-staining representing co-localized ricin and LRP1 (rows 3–4, right panel). Rows 1 and 2 in Fig. 7b display single-fluorophore controls for each antibody. Quantification of the relative amounts of colocalized ricin and LRP1, indicated that 91.2% of the cell-membrane bound ricin is linked to LRP1 (Fig. 7c), and that 43.5% of the LRP1 receptor is occupied with ricin.

To determine whether blocking the LRP1 receptor in the mice lung cells will prevent their intoxication by ricin, lung cells pretreated with anti-LRP1 antibody (1 hour, 37 °C) were exposed to ricin (10 ng/ml) and 48 hours later, cell viability was assessed utilizing the XTT assay. As shown in Fig. 7d, anti-LRP1 antibodies nearly completely protected the lung SCSs from ricin intoxication, with 96.5% of the cells that were pretreated with anti-LRP1 remaining viable, compared to 55.4% of the ricin-intoxicated cells which were not pretreated with the anti-LRP1 antibody. Pretreatment of the cells with a non-related antibody (anti-*bacillus anthracis*-PA antibody) did not provide any protection from ricin to lung SCSs. These findings confirm that LRP1 functions as an important receptor for ricin in lungs and that functional antagonism of the LRP1 receptor protects lungs from ricin intoxication.

## Discussion

The scientific literature dealing with ricin, provides detailed insights regarding the toxin’s crystallographic structure^17,18^, the unique mode of ricin intra-cellular trafficking^19–23^ as well as its catalytic activity mechanism^24,25^. In contrast, the binding profile of the toxin to the cell membrane has not been thoroughly configured and no specific receptor has yet been identified. The identification of specific receptors for ricin is of practical importance, as it may help in the development of specifically-tailored therapeutics that prevent the binding of the toxin to target cells.

So far, there has been a wide consensus as to the non-selective nature of ricin binding to all cell-surface glycoproteins or glycolipids containing a terminal *galactose* link^26–28^. Our observation that purified ricin was found to bind to a relatively low number of cell membrane proteins raised the possibility that, contrary to common belief, ricin interacts with specific transmembrane receptors. *Ricinus communis* agglutinin (RCA), which displays high-level homology to ricin, was found to have a wide binding profile. Indeed, the non-selective binding of RCA to cells is well documented and this protein is in fact used as an analytical tool for marking cell-membranes and for identifying oligosaccharide structures on cell surfaces^29,30^. Baenziger and Fiete^31^ have shown that RCA can bind glycopeptides with terminal *N-*acetylgalactosamine residues, while ricin cannot. This greater affinity of RCA was reported previously^32^, and may be related to the fact that RCA has double the number of binding sites, because of its tetramer structure, as opposed to ricin, which is a dimeric molecule^33^.

Mass spectrometry analysis revealed that ricin/cell-membrane-protein complexes contain either the mannose receptor or the prolow-density lipoprotein receptor-related protein 1. Both the ricin A and B chains carry glycoproteins that contain mannose-rich oligosaccharides and as such, ricin is known to bind to the mannose receptor on specific cells i.e., macrophages or non-parenchymal liver cells^34,35^. However, most cell types do not express the mannose receptor and consequently, ricin internalization into most cells is mediated exclusively by the B chain-related lectin activity^19,36^. In cells expressing the mannose receptor, competitive prevention of ricin binding can be completely achieved only in the presence of both mannose and galactose. In this case, the ricin molecule functions simultaneously as a lectin that recognizes and binds galactose moieties and as a mannose substrate that is recognized by target cell membranal mannose receptors. Quantification of the binding of ricin to Kupffer cells in the presence of mannose or galactose revealed that the binding of the toxin to mannose receptor constitute only 7% of the total binding^35^. Moreover, exposure of macrophages to transgenic ricin that is defective in both RTB binding sites was found to be non-toxic even when the binding was mediated by the mannose receptor^37^. Based on this finding, it seems that binding through RTB is essential for the toxicity of ricin, even in the presence of an alternative receptor. However, a recent study^38^ indicated an important role for mannose-receptor uptake of ricin by demonstrating that Kupffer cells could be equally protected from ricin intoxication by monoclonal antibodies directed against either the ricin A (non-lectin) or B (lectin) subunits. Thus, the mannose-receptor may play a greater role in ricin uptake than hitherto suggested, yet in parenchymal cells, which are mostly devoid of this receptor, an alternative point of entry is mandatory for ricin-induced cytotoxicity.

LRP1, a cell surface receptor belonging to the LDL receptor gene family^39^, which interacts with a variety of ligands (*e.g*. apolipoprotein E, α2-macroglobulin, amyloid precursor protein and several proteases and protease inhibitors^40–43^), plays a role in cell communication and signal transduction^40,44^ and functions also as the cell entry receptor for the *Pseudomonas exotoxin A* and the minor-group common cold virus^45,46^. This 600 kDa receptor is a type I single-pass transmembrane protein^47^ which contains a 515 kDa N-terminal extracellular heavy chain comprising 4 ligand-binding clusters, that is non-covalently attached to a 85 kDa membrane-integrated intracellular light chain. With the exception of receptor-associated protein (RAP), which serves as a molecular chaperone that interacts with all LRP1 clusters, most ligands bind exclusively to cluster II and/or IV^40^. The ability of LRP1 to bind with high affinity to numerous structurally distinct ligands, results from the presence of 31 ligand binding repeats in the molecule which form a unique contour surface and charge distribution, allowing multiple combinations of interactions between the ligand and receptor^48^.

In this study, functional blockage of LRP1 by three distinct and unrelated methods, indicated that this membrane-bound protein acts as the main host cell receptor for the ricin toxin. First, treatment with anti-LRP1 antibody prior to ricin intoxication reduced toxicity in a substantial manner. Second, addition of RAP in excess, reduced or even prevented ricin-induced intoxication of HEK293 cells. RAP is found primarily in the endoplasmic reticulum where it functions as a molecular chaperone^49^ that prevents association of newly synthesized LRP1 molecules with endogenous ligands. Due to its ability to antagonize ligand binding to this receptor, exogenously added RAP constitutes a powerful tool to study LRP1-mediated receptor/ligand interactions^50,51^. Finally, partial silencing of LRP1 expression in HEK293 cells by targeting siRNA, reduced ricin toxicity by 50%. From these results, we conclude that LRP1 is the primary endocytic receptor for ricin. We note in this context that the experimental procedures employed for functional blocking of LRP1 cannot refute the possibility that other components such as glycosylated ligands of LRP1, may play a role as intermediate molecules in ricin/LRP1 interactions. However since we provide ample evidence for the fact that functional blocking of the LRP1 receptor reduces ricin cytotoxicity, we firmly believe that the attribution of a major ricin target receptor role for LRP1 is fully valid.

The central role of LRP1 in ricin-induced HEK293 cell toxicity is in line with the fact that CHO cells were found to be resistant to *Pseudomonas exotoxin* (PE) as well as ricin^52,53^. LRP1 is known to be the membrane receptor of PE^44^ and the resistance of CHO cells to this bacterial toxin is not due to a paucity of LRP1 expression, but rather to the fact that the LRP1 receptor on this cell type is blocked by the RAP molecule^54^. The finding that LRP1 serves as a major receptor for ricin, provides an explanation for the resistance of CHO cells to the toxin and we assume that as in the case of PE, CHO refractivity towards ricin stems from the blockage of the LRP1 binding site by RAP. It should be noted that LRP1 was reported to be differentially glycosylated in a tissue specific manner, these differences affecting the receptor’s stability^55^. These variations in LRP1 glycosylation would probably affect the efficiency of LRP1-mediated ricin binding and uptake in different cells.

LRP1 contains cysteine-rich complement-type repeats (CRs), epidermal growth factor (EGF) repeats, β-propeller domains, a transmembrane domain and a cytoplasmic domain. The CR modules are organized into four highly conserved clusters (clusters I-IV^56^,). In this study we identified cluster II as the sole LRP1 binding site for ricin, and demonstrated that this binding is mediated by subunit B of the toxin. Cluster II and IV, which are responsible for the majority of ligand binding to the LRP1 receptor^40,57–59^ are highly similar in their binding properties, displaying only minor differences regarding their kinetics of interactions^60^. Huang *et al*.^61^, suggested that the CR modules within these clusters, present different charge densities and hydrophobic patches, which in turn lead to varying receptor-ligands interactions responsible for the different ligand specificity of each cluster. Certain ligands, such as ricin, recognize different combinations of the CRs located within a single binding cluster (cluster II), whereas others, such as alpha-2-macroglobulin, were found to bind to CRs located on different clusters (clusters II and IV). Identification of the CR regions on the LRP1 that are important for binding ricin, will allow developing a specific inhibitor capable of preventing this interaction.

The binding kinetics of ricin to cluster II were found to fit a 2:1 heterogeneous ligand model, in line with the fact that ricin has two lectin-binding domains within the B-subunit. The overall affinities values (K_D_) of the two binding sites of ricin 81 and 47 nM (K_D_1 and K_D_2, respectively) support the assumption that the two ricin-lectin binding sites interact with LRP1 in an independent manner. These K_D_ values, which are two orders of magnitude higher than the affinity interactions commonly measured between lectins and oligosaccharides (normally in the millimolar range^62^,), can explain the predilection of ricin binding to the LRP1 receptor, rather than to other galactose residues on the cell surface.

Ricin can bind to free galactose, as attested by the fact that toxin binding to LRP1 cluster II was abolished in the presence of excess of galactose. Nevertheless, the marked proclivity of ricin towards LRP1, as opposed to other cell-surface glycoproteins, clearly indicates that the toxin’s interaction with glycans is strongly influenced by vicinal non-sugar structural elements. The interactions of other lectin toxins, were also found to be restricted to specific receptors, *i.e*. Shiga and Cholera toxins to the Gb3 and GM1 receptors, respectively^63,64^.

Finding that LRP1 plays a major role in the intoxication process of ricin, it was of interest to determine whether our monoclonal neutralizing antibodies^15,16^ impair the binding of ricin to LRP1. We found that anti-ricin MH77 monoclonal antibody reduces binding of ricin to cluster II by more than 70%, whereas other anti-ricin monoclonal antibodies, MH1, MH73 and MH75 did not prevent binding of the toxin to this cluster, even though two of them, MH73 and MH75, bind to non-overlapping epitopes located on the surface of RTB^15^. Though these 4 antibodies conferred similar survival rates to ricin-intoxicated mice when administered six hours post exposure, when mice were treated 24 hours post exposure, antibody MH77 provided significantly higher protection than the other 3 antibodies^16^. The fact that the most effective anti-ricin monoclonal antibody is the one that prevents the binding of ricin to LRP1, underscores the pivotal role of the LRP1 receptor in ricin intoxications.

The toxicity of ricin depends on the route of exposure, pulmonary exposure being considered most dangerous^65^. Pathological studies of pulmonary ricin intoxications demonstrated that injury is confined to the lungs as manifested by perivascular, interstitial and alveolar edema, influx of neutrophils to the lungs and the mounting of an acute inflammatory response. Flooding of the lungs leads to respiratory insufficiency and death^66^. To probe the possible role of the LRP1 receptor in ricin-mediated pulmonary poisoning, we tested the contribution of LRP1 to ricin intoxication in a single cell suspension produced from mice lungs. Quantitation of LRP1 expression in different cell populations of the lungs, revealed a strong correlation between LRP1 expression levels in the different cell subtypes and ribosomal damage levels, i.e. 28 S rRNA depurination, in these cells following pulmonary exposure to ricin. This finding strongly suggests that the expression of LRP1 by lung cells dictates their sensitivity towards ricin. Moreover, confocal microscopy imaging of intoxicated lung SCSs revealed that over 80% of the ricin is linked to LRP1. Most importantly, treating those SCS with anti-LRP1 antibody prior to ricin exposure prevented their intoxication. These results demonstrates beyond doubt the importance of this receptor in pulmonary ricin intoxications.

Studies carried out in our laboratory on a swine model for pulmonary ricinosis, demonstrated that the pathological state ensuing pulmonary exposure to ricin is that of acute respiratory distress syndrome (ARDS)^67^. Cumulative evidence suggests that the ectodomain of LRP1 is proteolytically cleaved from cell surfaces in different clinical pathologies including ARDS, releasing a soluble form of this receptor (sLRP1)^68–71^. sLRP1 maintains the ligand binding characteristics of cell-bound LRP1 and may therefore act as a competitive inhibitor of ligand binding and clearance by cell surface-associated LRP1. We now intend to assess whether LRP1 shedding occurs in lungs of animals exposed to ricin, and if so, whether this shedding of the main receptor for ricin has a beneficial or harmful contribution to pulmonary ricinosis.

The present study has demonstrated for the first time that a plant toxin can act by binding to a receptor known to mediate binding and uptake of physiological ligands. Ricin toxin is capable of killing cells of many different animal species and of various tissues. In order to be broadly effective as a virulence factor it must make use of widely distributed and highly conserved molecules, such as the LRP1 receptor. We conclude that ricin is one of several ligands which use LRP1 to enter cells and that cells displaying this receptor on their surface are likely to be targets for its toxic effects.

## Materials and Methods

### Ricin and ricinus communis agglutinin (RCA) preparations

Crude ricin was prepared from seeds of endemic *Ricinus communis*, essentially as described before^72^. Briefly, seeds were homogenized in a Waring blender in 5% acetic acid/phosphate buffer (Na_2_HPO4, pH 7.4) the homogenate was centrifuged and the clarified supernatant containing the toxin was subjected to ammonium sulfate precipitation (60% saturation). The precipitate was dissolved in PBS and dialyzed extensively against the same buffer.

For the preparation of purified ricin and *Ricinus communis agglutinin* (RCA), crude ricin was loaded consecutively onto 2 columns, the first column contains activated Sepharose which binds and thereby depletes the RCA. RCA bound to the Sepharose column was thereby purified following elution with 0.5 M Galactose in PBS. The flow-through of the activated Sepharose column (crude ricin) was loaded onto the second column, containing α-lactose (lactamyl) agarose (Sigma-Aldrich, Rehovot, Israel), and the column was washed in order to discard non-related impurities. Purified ricin was eluted from the lactamyl agarose column with 0.5 M Galactose in PBS.

### Human Embryonic Kidney 293 - acetylcholinesterase cells

Human Embryonic Kidney 293 (HEK293)- Acetylcholinesterase (AChE) cells^12^ were cultured in Dulbecco’s modified Eagle’s medium (DMEM) (Biological Industries, Beit Haemek, Israel) supplemented with 10% fetal calf serum (FCS). For the cytotoxicity studies, cells were seeded in 96-well plates (1 ×10^5^ cells/well) and exposed to ricin for 1 hour in the presence or absence of rabbit anti-LRP1 antibodies directed against the N or C terminals (L2295 and L2170, Sigma–Aldrich, Ness-Ziona, Israel) or recombinant human Receptor-associated Protein (RAP) (10 nM, Enzo Life Sciences, Lausen, Switzerland). Cells were then rinsed and refed with fresh DMEM and incubated for 18–24 hours (37 °C, 5% CO_2_) and then incubated for 2 hours in fresh medium. Secreted AChE was determined according to Ellman *et al*.^73^, in the presence of 0.1 mg/ml bovine serum albumin (BSA), 50 mM sodium phosphate buffer (pH 8.0), 0.5 mM acetylthiocholine iodide (ATC) and 0.3 mM 5,5’-dithiobis-2-nitrobenzoic acid (Sigma-Aldrich). The assay was carried out at 27 °C and monitored by a ThermoMax microplate reader (Molecular devices, Ramsey, MN, USA).

### Animals

Animal experiments were performed in accordance with the Israeli law and approved by the Ethics Committee for Animal Experiments at the Israel Institute for Biological Research (Project Identification Codes M-12–15, M-9–19). Treatment of animals was in accordance with regulations outlined in the U.S. Department of Animal Welfare Act and the conditions specified in the National Institute of Health’s Guide for Care and Use of Laboratory Animals. All animals in this study were female CD-1 mice (Charles River, Margate, UK) weighing 27–32 g. Mice were housed in filter-top cages in an environmentally controlled room, maintained at 21 ± 2 °C and 55 ± 10% humidity and had access to food and water *ad libitum*. Lighting was set to mimic a 12-hours:12-hours dawn-dusk cycle.

### Extraction of lung cell membranes

Lungs were harvested from terminally anesthetized mice (ketamine, 1.9 mg/mouse and xylazine, 0.19 mg/mouse) which were subjected to PBS perfusion *via* the heart. Lungs were then homogenized in TE buffer (100 mM Tris and 10 mM EDTA PH 7.5) containing 15% sucrose solution and centrifuged at 10,000 g for 30 minutes. The supernatant was ultracentrifuged at 100,000 g for 120 minutes at 4 °C to pellet the total membranes. The membrane pellet was dissolved in 3 ml of TE 40% sucrose solution. Continuous sucrose gradients were prepared by layering sucrose solutions 20–50% (prepared in TE buffer) into 14 ×89 mm ultracentrifuge tubes (Beckman, Indianapolis, IN, USA) including the membrane pellet dissolved in 40% sucrose. The membrane-pellet-containing sucrose gradients were ultracentrifuged at 100,000 g over night at 4 °C. The crude cell-membrane fraction located in the middle of the tubes, was collected with a syringe and washed three times with PBS.

### Western blot analysis

Isolated lung membranes or HEK293 cell lysate were heated to 95 °C in Laemmli sample buffer (Bio-Rad, Jerusalem, Israel) for 5 minutes. Lysates were resolved by 4–12% Bis-Tris (for lung membranes) or 3–8% Tris-Acetate sodium dodecyl sulfate-polyacrylamide gel electrophoresis (SDS-PAGE, for HEK293 cell lysate) (Invitrogen, Carlsbad, CA, USA) and transferred onto nitrocellulose membranes (Invitrogen). Membranes were blocked with 5% nonfat blotting grade blocker (170–6404; BioRad, Hercules, CA, USA) in tris-buffered saline/Tween-20 for 1 hour at room temperature. For immunoblotting of lung cells-membranes, the membranes were incubated with purified ricin or RCA (20 µg/ml) for 2 hours, washed and incubated with rabbit-anti-ricin polyclonal antibody for 2 hours. For immunoblotting of HEK293 cell lysate, the membranes were incubated with rabbit anti-LRP1 C terminal antibody (L2170, Sigma–Aldrich) and rabbit anti-actin (A2066, Sigma-Aldrich) over night at 4 °C. All membranes were incubated with anti-rabbit IgG horseradish peroxidase-linked antibody (A0545, Sigma-Aldrich) for 1 hour, developed and band intensities were quantified as previously described^74^.

### Blue Native gel electrophoresis

Membranes of mice lung cells were incubated with purified ricin for 2 hours, rinsed with PBS and centrifuged at 5000 RPM for 10 minutes. The pellet was solubilized with 2% dodecyl maltoside and resolved by Blue-Native PAGE^75^ on 4–16% gradient gels (NativePage, Novex, ThermoFischer, Waltham, MA, USA) together with native high molecular weight markers (Amersham Biosciences, Bath, UK) on a BioRad protean II minigel system. Gels were run at 35 V for 30 min and at 350 V for 3 hours. After electrophoresis, half of the gels were transferred onto a PVDF membrane (Invitrogen). Membranes were blocked with 5% nonfat blotting grade blocker (170–6404; BioRad) in tris-buffered saline/Tween 20 for 1 hour at room temperature. For immunoblotting of lung cells-membranes, the membranes were incubated with rabbit-anti-ricin polyclonal antibody for 2 hours and then with anti-rabbit IgG horseradish peroxidase-linked antibody (Sigma-Aldrich) for 1 hour, developed with Clarity Western ECL Substrate (BioRad) and visualized by a chemiluminescence detection system (Fujifilm, LAS3000).

The remaining membranes were stained with colloidal Coomassie stain (SimplyBlue, Invitrogen). From the stained gels, 3 positive bands for ricin (as visualized on the PVDF membranes) were excised with a scalpel and destained for further mass spectrometric analysis. Proteins in each band were reduced with 5 mM dithiothreitol and alkylated with 10 mM iodoacetamide (Sigma-Aldrich) in the dark for 30 min at 21 °C. Proteins were digested by rehydrating the gel pieces with 12.5 ng/µl trypsin (Promega, Madison, WI, USA) in 25 mM NH_4_HCO_3_ at 4 °C for 10 min following by overnight incubation at 37 °C. Peptides were then extracted by addition of 50% (vol/vol) acetonitrile 5% (vol/vol) formic acid, vortex, sonication, centrifugation, and collection of the supernatant were performed. Samples were dried and stored at −80 °C until further analysis.

### Liquid chromatography and mass spectrometry

Liquid chromatography and mass spectrometry were performed by the de Botton Institute for Protein Profiling at The Nancy and Stephen Grand Israel National Center for Personalized Medicine (Weizmann Institute of Science, Rehovot, Israel) as previously described^76^. For identification purposes, raw data was first processed using Proteome Discoverer v1.41. MS/MS spectra were searched using Mascot v2.4 (Matrix Sciences, Chicago, IL, USA) and Sequest HT. Data were searched against *Mus musculus* protein database as downloaded from UniprotKB (http://wwww.uniprot.org/).

### Immunolocalization of ricin and LRP1 by confocal microscopy

Anti-ricin Ab was conjugated (1 mg) with the Alexa Fluor 594 protein labeling kit (Molecular Probes, Thermo Fisher Scientific, Waltham, MA, USA) according to the manufacturer’s instructions.

For immunolocalization experiments, HEK293 cells or a single cell suspension (SCS) from mice lungs were seeded on #1 glass cover slips in 24-well dishes and exposed to ricin (100 ng/ml). Cells were fixed with 4% paraformaldehyde (PFA, Gadot, Israel) for 10 min at 4 °C, washed three times with PBS, and placed for 1 hour in a blocking solution (10% normal goat serum (NGS)) in PBS containing 0.05% Tween-20 (P5927, Sigma-Aldrich). Cells were incubated in a 1:500 dilution of rabbit anti-LRP1 Alexa Fluor 488 (Abcam, Cambridge, MA, USA) and anti-ricin Alexa Fluor 594 in an antibody cocktail solution (50% blocking solution/0.05% Tween-20/PBS) for 24 hours at 4 °C. Cover slips were processed as described previously^77^ and imaged in a sequential manner using an LSM 710 confocal scanning microscope (Zeiss, Jena, Germany) equipped with following lasers: argon multiline: 458/488/514 nm); diode: 405 nm; DPSS: 561 nm; and heliumneon: 633 nm. The percent of colocalization was quantified using Zen software (version 2.1, 2008; Zeiss). Image parameters: Scan mode-plane; Dimensions- X:1691 Y:1127 8-bit; Average-8; Pixel dewl-1.27 µs.

### XTT assay for cell viability

Lung SCS were seeded onto a 96 well plate (50,000 cells/well) and incubated (37 °C, 5% CO_2_) in DMEM (Biological Industries) for 6 hours to enable cell adhesion and spreading. Cells were treated with anti-LRP1 antibodies (L2295 and L2170, Sigma–Aldrich) or with control anti-*bacillus anthracis* protective antigen (PA) antibody 1 hour prior to ricin exposure (10 ng/ml) for 48 hours. Tetrazolium salt (XTT, Biological Industries, 50 µl/well) was added to each well, plates were incubated at 37 °C in the dark for 6 hours and colorimetric changes were measured using a ThermoMax microplate reader (Molecular devices, Ramsey, MN, USA) at 475 nm. The viability in each well was compared with that of the non-intoxicated control wells.

### siRNA knockdown of LRP1 in HEK293 cells

Single stranded, LRP1-specific sense and antisense RNA oligonucleotides were synthesized by Ambion (Austin, TX, USA). For transfection, cells were trypsinized and seeded into 96 well plates (1 ×10^5^ cells/well) in DMEM. After 24 hours, cells were transfected with siRNA using Lipofectamine 2000 (Invitrogen) according to the manufacturer’s specifications. Cells were harvested for analysis or exposed to ricin 48 hours after transfection.

### Reverse transcription and RT-PCR

Total RNA, isolated from transfected HEK293 cells (RNeasy Mini Kit, Qiagen, Valencia, CA, USA) was eluted in nuclease-free water and stored at −80 °C. Reverse transcription and quantitative real-time PCR were performed as previously described^78^. Specific primers and fluorescent TaqMan probe for LRP1 were selected from a list of predesigned assays (Assays-on-demand LRP1, Hs 00233856, Applied Biosystems). HPRT1 (Hs99999909, Applied Biosystems) was used as a control housekeeping gene. Expression levels were measured in triplicate and threshold cycle (Ct) values were normalized to the housekeeping gene.

### Binding studies and affinity measurements

Binding studies were carried out using the Octet Red system (ForteBio, Version 8.1, Menlo Park, CA, USA) as previously described^15^. Briefly, streptavidin-coated biosensors were loaded with biotinylated LRP1 clusters (II, III, IV, R&D) (5 µg/mL) for 300 s followed by a wash. The sensors were then reacted for 300 seconds with increasing concentrations of ricin (2.5–20 µg/ml) in the presence or absence of monoclonal anti-ricin antibodies (MH1, MH73, MH75, MH77)^15^ and then moved to buffer-containing wells for another 300 seconds or up to several hours (dissociation phase). Binding (300 seconds to reach saturation) and dissociation (300 seconds) were measured as changes over time in light interference after subtraction of parallel measurements from unloaded biosensors. Sensorgrams were fitted with a 2:1 heterogeneous ligand binding model using the Octet data analysis software 8.1 (Fortebio, Menlo Park, CA, USA). Values are presented as an average of several repeated measurements.

### Preparation of single cell suspension from mice lungs

Mice were terminally anesthetized with intraperitoneal injection of ketamine (1.9 mg/mouse) and xylazine (0.19 mg/mouse)) followed by heart perfusion with PBS. Harvested lungs were minced into small pieces, which were then subjected to enzymatic digestion with 4 mg/ml collagenase D (Roche, Mannheim, Germany) in PBS containing Ca^+2^ and Mg^+2^ (Biological Industries, Beit Haemek, Israel) for 2 hours at 37 °C. The tissue was then meshed through a 40 μm cell strainer and centrifuged at 300 g for 5 minutes. Cells were suspended in DMEM (Biological Industries) and incubated in 37 °C at 5% CO_2_.

### Analysis of lung cells by flow cytometry

Lungs were harvested, minced into small pieces and digested for 2 hours at 37 °C with 4 mg/ml collagenase D (Roche, Mannheim, Germany) in PBS containing Ca^+2^ and Mg^+2^ (Biological Industries, Beit Haemek, Israel). The tissue was then meshed through a 40 μm cell strainer and red blood cells were lysed with ACK lysis buffer (150 mM NH4Cl, and 10 mM KHCO3). Cells were stained in flow cytometry buffer (PBS with 2% FCS, 0.1% sodium azide and 5 mM EDTA) using anti-LRP1 Alexa Fluor 594 antibody and cell surface antibodies as we published previously^7^. Cells were analyzed by using FACSCalibur (BD Biosciences, San Jose, CA,USA)) and FlowJo software (VERSION 7.1.2, Tree Star, Ashland, OR, USA) according to the following markers: neutrophils, CD11c^-^ and Gr-1^high^; alveolar macrophages, autofluorescence, CD11c^high^, Gr-1^int^, endothelial cells, CD45^-^ and CD31^+^ and epithelial cells, CD45^-^, CD31^-^, CD326^+^.

### Statistical analysis

Simple comparisons were performed using the unpaired two-tailed Student’s t-test. For multiple comparisons, oneway ANOVA with Bonferroni’s multiple comparison test was applied. Significance was set at P < 0.05. Statistical analysis was calculated using Prism software (version 5.01, 2007; GraphPad Software, La Jolla, CA). All data are presented as means ± SE.

## Supplementary information

Supplementary Information.

## Data Availability

All datasets generated during the current study are available from the corresponding author upon reasonable request.
