# Supplementary material for: The low density receptor-related protein 1 plays a significant role in ricin-mediated intoxication of lung cells
Source: Sci Rep. 2020 Jun 2;10:9007. doi: 10.1038/s41598-020-65982-2 (PMC7265403; doi:10.1038/s41598-020-65982-2)
Supplement: Supplementary file 1 — Supplementary Information. [file 41598_2020_65982_MOESM1_ESM.pdf]

The low density receptor-related protein 1 plays a significant role in ricin-mediated intoxication of lung cells

Reut Falach[1*], Anita Sapoznikov[1], Yoav Gal[1], Eytan Elhanany[1], Yentl Evgy[1], Ohad Shifman[1], Moshe Aftalion[1], Sharon Ehrlich[1], Shlomi Lazar[2], Tamar Sabo[1], Chanoch Kronman[1], Ohad Mazor[3]

Supplementary data file:

Figure S1:

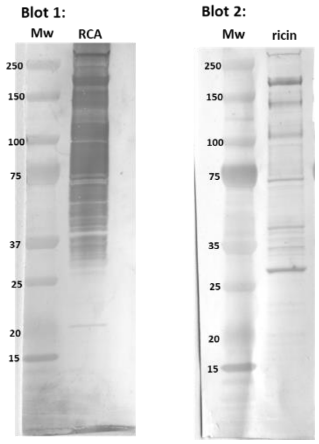

Figure S1: Lectin blot of membrane-bound proteins from mice lungs (source for Figure 1) : Lung cell membrane proteins were resolved by SDS-PAGE, transferred to absorbent membranes, and incubated with purified preparations of RCA (blot 1) or ricin (blot 2).

Native gel and blot (supplement to Table 1)

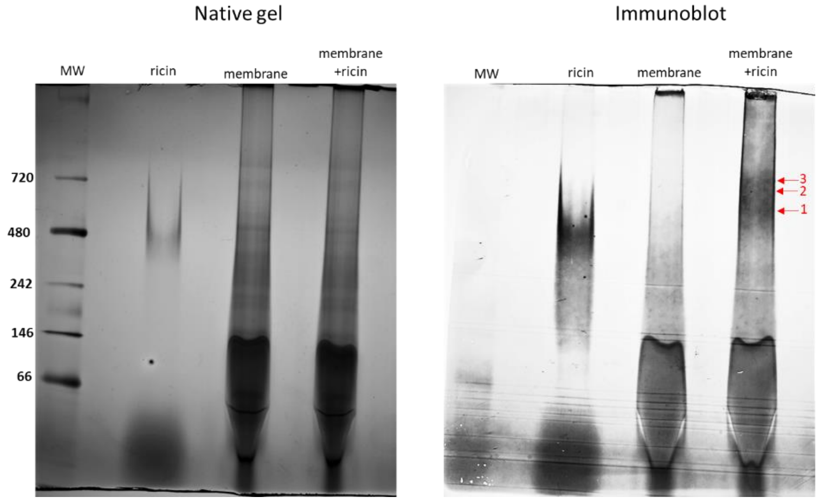

Native gel and immunoblot of lung membranes exposed to ricin: Lung cell membranes incubated with ricin (membrane + ricin) or not (membrane), were resolved on native gels and transferred to absorbent (PDVF) membranes which were then labeled with polyclonal anti-ricin antibodies. The 3 high molecular weight bands that were discerned on the immunoblot (red arrows) served as a guide for excision of ricin/membranal protein complexes from the native gel. The excised gel bands were processed by in-gel digestion and then subjected to mass spectrometry analysis.

Figure S4b:

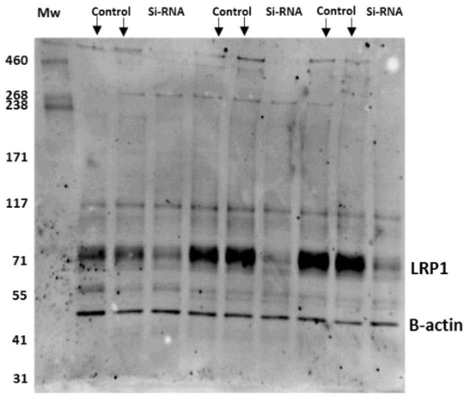

Figure S4B: LRP1 silencing in HEK293-AChE cells (source for Figure 4b). Western blotting analysis of HEK293-AChE cells proteins following treatment with LRP1 siRNA (3 repeats). Control lanes (two lanes for each sample, 3 repeats) refer to cells treated with non-sense siRNA. Anti-actin blot was used as a loading control.
